# Integrating Hypoxia Signatures from scRNA-seq and Bulk Transcriptomes for Prognosis Prediction and Precision Therapy in Cervical Squamous Cell Carcinoma and Endocervical Adenocarcinoma

**DOI:** 10.3390/ijms26031362

**Published:** 2025-02-06

**Authors:** Kexin Yu, Shibo Zhang, Jiali Shen, Meini Yu, Yangguang Su, Ying Wang, Kun Zhou, Lei Liu, Xiujie Chen

**Affiliations:** Department of Pharmacogenomics, College of Bioinformatics Science and Technology, Harbin Medical University, Harbin 150081, China; ykx@hrbmu.edu.cn (K.Y.); 2023020558@hrbmu.edu.cn (S.Z.); 2018158020@hrbmu.edu.cn (J.S.); ymn2549700843@163.com (M.Y.); 2021020532@hrbmu.edu.cn (Y.S.); 2022020545@hrbmu.edu.cn (Y.W.); 2021020565@hrbmu.edu.cn (K.Z.)

**Keywords:** hypoxia, cervical squamous cell carcinoma and endocervical adenocarcinoma, risk model, scRNA-seq, precision medicine

## Abstract

Hypoxia, a common feature in many malignancies, is particularly prominent in cervical squamous cell carcinoma and endocervical adenocarcinoma (CESC). Investigating the mechanisms underlying hypoxia is essential for understanding the heterogeneity of CESC and developing personalized therapeutic regimens. Firstly, the CESC-specific hypoxia gene sets shared between single-cell RNA sequencing (scRNA-seq) and bulk data were identified through Weighted Gene Correlation Network Analysis (WGCNA)and FindMarkers analyses. A CESC-specific hypoxia-related score (CSHRS) risk model was constructed using the least absolute shrinkage and selection operator (LASSO)and Cox regression analyses based on these genes. The prognostic differences were analyzed in terms of immune infiltration, mutations, and drug resistance. Finally, a nomogram model was constructed by integrating clinicopathological features to facilitate precision treatment for CESC. This study constructed a CSHRS risk model that divides patients into two groups, and this model can comprehensively evaluate the tumor microenvironment characteristics of CESC, provide accurate prognostic predictions, and offer rational treatment options for patients.

## 1. Introduction

The prognosis of cervical squamous cell carcinoma and endocervical adenocarcinoma (CESC) patients is influenced by diagnostic accuracy as well as recurrence and metastasis occurrence [1]. While current clinical screening methods and HPV testing have been highly successful, challenges such as false positives, false negatives, and limited ability to predict disease progression remain [2,3]. Therefore, accurate prediction and timely adjustments to treatment strategies are crucial for improving patient therapy outcomes.

Hypoxia, a hallmark in CESC, is a key driver of recurrence and metastasis [4,5,6,7]. It is critical in promoting tumor growth, invasion, and metastasis by regulating essential processes such as angiogenesis, metabolic shifts, and immune evasion [8,9,10,11,12]. The effects of hypoxia are further intensified by its ability to enhance resistance to radiotherapy and chemotherapy, leading to higher rates of treatment failure [13]. Investigating the mechanisms of hypoxia in CESC is vital for enhancing our understanding of CESC heterogeneity, optimizing therapeutic strategies, and ultimately improving patient prognosis.

Bulk data provide an overall characterization of disease tissues, but tumor tissues consist of various cell types, each playing different roles in tumor initiation, progression, and prognosis [14]. Therefore, relying solely on bulk data to identify prognostic markers cannot accurately reflect the contributions of different cell types within the tumor and the dynamic changes in the tumor microenvironment or cell-cell interactions [15]. This limitation can dilute or mask signals from critical cell subsets, such as immune or stromal cells, thereby reducing the biological interpretability and reliability of the model [16,17]. The rapid advancement of single-cell RNA sequencing (scRNA-seq) technology addresses this limitation by providing expression profiles at the single-cell level [18,19]. Integrating bulk and scRNA-seq data uses the breadth of bulk data and the depth of single-cell data, enhancing the predictive power and robustness of models. Therefore, this study aims to delineate the hypoxia landscape of CESC integrating hypoxia signatures derived from scRNA-seq and bulk data, providing a valuable suggestion for prognostic stratification and personalized treatment of CESC. Using machine learning algorithms (Supplementary Figure S1), we constructed a CESC-specific hypoxia-related score (CSHRS) risk model based on the identified hypoxia signatures. To investigate the different prognoses of CSHRS groups, we conducted comprehensive analyses encompassing immune infiltration, trajectory analysis, mutation analysis, and drug sensitivity using scRNA-seq and bulk data. Additionally, a nomogram was constructed to facilitate personalized treatment strategies for CESC patients.

## 2. Results

### 2.1. Hypoxia Is a Primary Prognosis Risk Factor in CESC

**Figure 1:**
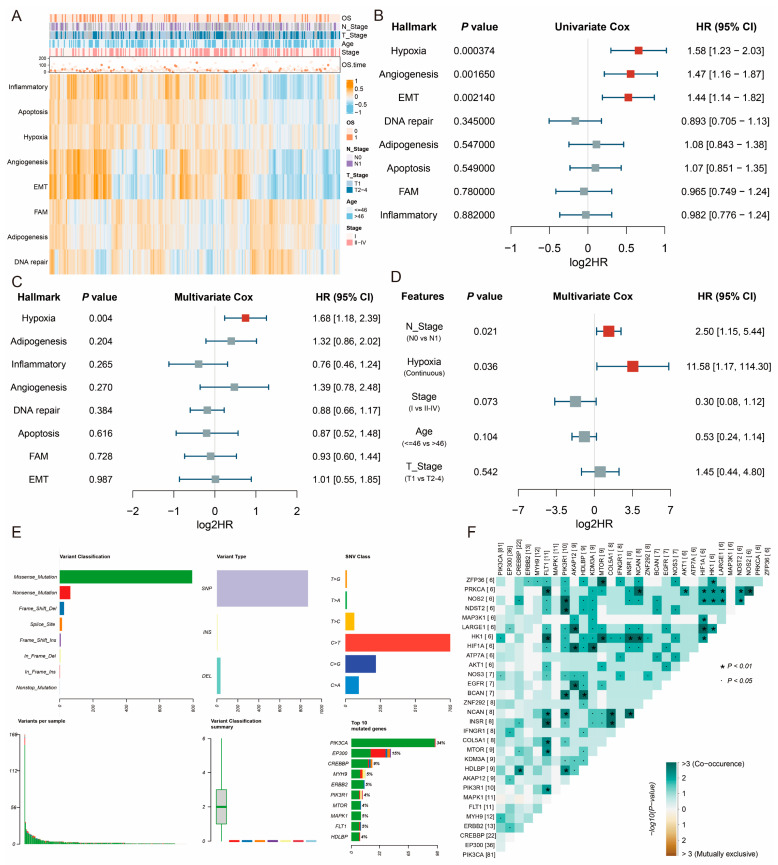
Hypoxia is a primary prognosis risk factor in cervical squamous cell carcinoma and endocervical adenocarcinoma (CESC). (**A**) Correlation analysis between hallmarks and clinicopathological features. (**B**) Univariate Cox regression analysis among various hallmarks of cancer. (**C**) Multivariate Cox regression analysis among various hallmarks of cancer. (**D**) Multivariate Cox regression analysis between hypoxia hallmark and clinicopathological features. (**E**) Somatic mutation analysis of 282 hypoxia hallmark genes. (**F**) Co-mutation analysis among hypoxia hallmark genes. The [XX] represents the number of samples with gene mutations.

To evaluate the impact of various hallmarks of cancer defined by Hanahan [20] in cervical squamous cell carcinoma and endocervical adenocarcinoma (CESC) prognosis, a heatmap was generated to show the correlation between hallmarks and clinicopathological features based on their single-sample gene set enrichment analysis (ssGSEA) Z-score matrix (Figure 1A, Supplementary Tables S1 and S2). We observed that “Inflammatory”, “Apoptosis”, “Hypoxia”, “Angiogenesis”, and “Epithelial-mesenchymal transition (EMT)” were more strongly associated with poor prognosis than other hallmarks (Figure 1A). A univariate Cox regression analysis quantified the impact of hallmarks on patient prognosis. It demonstrated that “Hypoxia”, “Angiogenesis”, and “EMT” were significantly correlated with poor prognosis, with “Hypoxia” showing the most significant *p-value* (Figure 1B). A multivariate Cox regression analysis showed that hypoxia was the only significant prognostic risk factor among hallmarks (Figure 1C). In the multivariate Cox regression analysis combined with clinicopathological features, it was found that “Hypoxia” remained a significant prognostic risk factor (Figure 1D). A somatic mutation analysis of 282 hypoxia hallmark genes identified mutations in 168 samples, primarily as missense mutations, which indicate alterations in protein function (Figure 1E). A few genes exhibited particularly high mutation frequencies (Figure 1E). A subsequent correlation analysis revealed extensive co-mutation among these genes, suggesting that interactive relationships might collectively contribute to oncogenic pathways or molecular mechanisms enhancing cancer cell adaptability or malignant potential (Figure 1F). All these findings above emphasize hypoxia as a primary prognostic risk factor in CESC and highlight the importance of integrating hypoxia-associated gene analysis for patient prognosis assessment.

### 2.2. Identification of CESC-Specific Hypoxia-Related Genes

**Figure 2:**
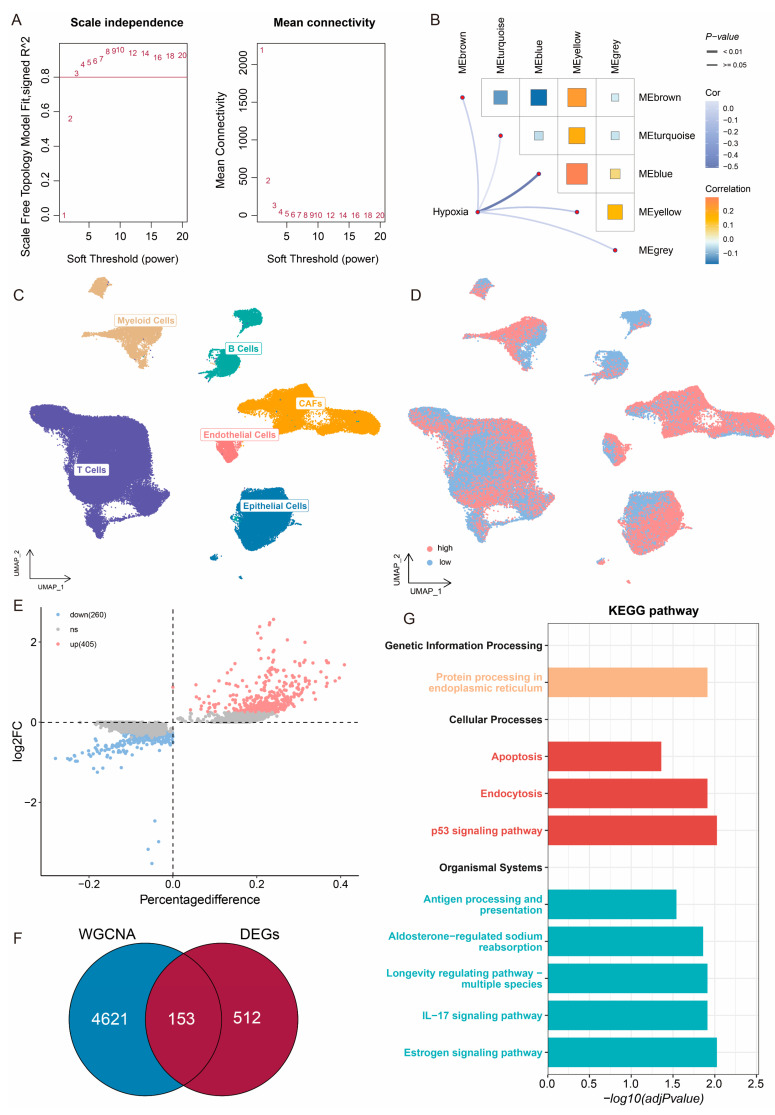
Identification of CESC-specific hypoxia-related genes. (**A**) The nature of the network topology constructed with unique power values. (**B**) The correlation between different modules and hypoxia. (**C**) UMAP plot showing the annotation for five cell types. (**D**) Grouping of cells by hypoxia-related genes. (**E**) Volcano plot of differentially expressed genes. (**F**) Venn plot showing the hub genes intersected by WGCNA and DEGs. (**G**) KEGG functional enrichment analysis of CSHRGs.

Considering the cumulative impact of hypoxia on CESC prognosis through the cooperative action of multiple genes, we comprehensively identified a hypoxia-associated gene set specific to CESC at both the bulk and single-cell RNA sequencing (scRNA-seq) levels. Firstly, Weighted Gene Correlation Network Analysis (WGCNA)was performed with bulk data and hypoxia ssGSEA Z-score to construct a scale-free co-expression network for identifying gene modules associated with hypoxia. Five gene modules were generated with a power of 3 as the optimal soft threshold (Figure 2A,B). Pearson tests were employed to assess correlations between hypoxia and modules. Notably, the blue module (4774 genes in total) demonstrated the strongest correlation with the hypoxia (Figure 2B). Subsequently, 130,316 cells were classified into 13 clusters at the scRNA-seq level using the FindClusters function (resolution = 0.1). From these clusters, five major cell types were identified via classical cell markers, including T cells (*CD3E, CD8A, CD3D*), B cells (*CD19, MS4A1, CD79A*), myeloid cells *(CD14, CD68, LYZ*), endothelial cells (*PECAM1, VWF, CDH5*) and cancer-associated fibroblasts (CAFs) (*ACTA2, FAP, PDGFRB*) (Figure 2C). The hypoxia score was calculated for each cell using the AUCell package, and all cells were classified into high- and low-hypoxia cells according to the median score (Figure 2D). The FindMarkers function obtained differentially expressed genes (DEGs) between high- and low-hypoxia cells, including 665 genes (Figure 2E). The blue modular genes were intersected with the DEGs, and a total of 153 genes related to hypoxia were obtained and were considered as CESC-specific hypoxia-related genes (CSHRGs) (Figure 2F).

KEGG (Kyoto Encyclopedia of Genes and Genomes) is a widely used bioinformatics database that provides comprehensive information on genes, proteins, metabolic pathways, drugs, and diseases. In this study, we conducted a KEGG pathway enrichment analysis to explore the role of CSHRGs in tumorigenesis and development (Figure 2G). The results reveal that 153 CSHRGs were significantly enriched across nine non-disease-related pathways, which were further classified into three main categories (Figure 2G). The first category, Genetic Information Processing, includes the Protein processing in endoplasmic reticulum pathway. Under hypoxic conditions, endoplasmic reticulum stress increases, triggering the unfolded protein response, which helps tumor cells survive in adverse microenvironments [21,22]. The second category, Cellular Processes, involves the p53 Signaling, Endocytosis, and Apoptosis pathways. Under hypoxic conditions, cancer cells typically suppress the p53 function to avoid apoptosis and facilitate nutrient uptake and signaling regulation through endocytosis. Moreover, the activation of hypoxia-related genes can induce selective apoptosis, contributing to therapeutic resistance [23,24,25,26,27]. The third category, Organismal Systems, includes the Estrogen Signaling, IL-17 Signaling, and Aldosterone-Regulated Sodium Reabsorption pathways. Hypoxia influences hormone and immune signaling, promoting inflammation and immune evasion, which collectively provide a favorable environment for tumor progression [28,29,30,31]. In summary, our study identifies these 153 CSHRGs, which collectively impact patient prognosis regulation through the modulation of various pathways supporting tumor cell survival, proliferation, and immune evasion.

### 2.3. Construction and Validation of the CSHRS Risk Model

**Figure 3:**
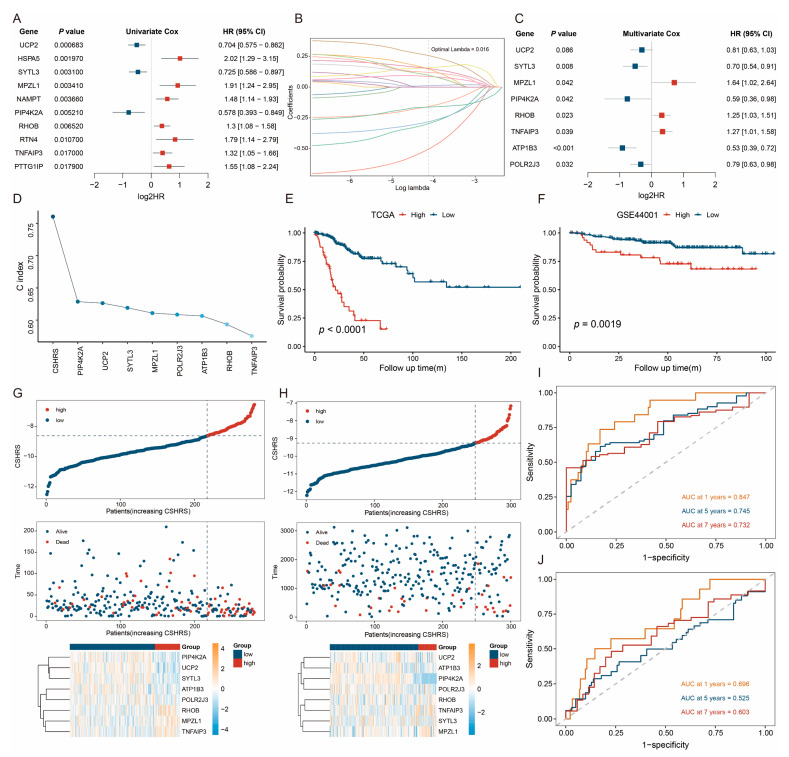
Construction and validation of the CESC-specific hypoxia-related score (CSHRS)risk model. (**A**) Univariate Cox regression analysis of CSHRGs. (**B**) Coefficient distribution plots of log (lambda) sequences. (**C**) Multivariate Cox regression analysis of CSHRGs. (**D**) C-index of CSHRS model and model genes. (**E**,**F**) TCGA: (**E**) GSE44001, (**F**) Kaplan–Meier survival curves. (**G**,**H**) TCGA: (**G**) GSE44001 (**H**) CSHRS distribution, survival status, and gene expression patterns for patients in high- and low-CSHRS groups. (**I**,**J**) TCGA: (**I**) GSE44001, (**J**) time-dependent ROC analysis.

To analyze the comprehensive impact of CSHRGs on the progression of CESC patients, we constructed a CESC-specific hypoxia-related score (CSHRS)risk model to assess patient prognosis. Firstly, 19 CSHRGs significantly associated with OS were identified using univariate Cox proportional regression analysis (Figure 3A). These genes took the least absolute shrinkage and selection operator (LASSO)analysis for dimensionality reduction (Figure 3B), and 14 genes were selected. After the multivariate Cox regression analysis, only eight genes remained (Figure 3C). The β coefficients and gene expression levels from the multivariate Cox analysis were used to establish the CSHRS model: CSHRS = exp (*MPZL1*) × 0.496 + exp (*RHOB*) × 0.219 + exp (*TNFAIP3*) × 0.235 − exp (*ATP1B3*) × 0.638 − exp (*POLR2J3*) × 0.239 − exp (*UCP2*) × 0.214 − exp (*SYTL3*) × 0.35 − exp (*PIP4K2A*) × 0.524. The predictive performance of the CSHRS was evaluated using the C-index from the Cox regression model (Figure 3D). Compared to the univariate Cox regression analyses of individual genes, CSHRS demonstrated the highest C-index, indicating superior predictive capability (Figure 3D). Based on the CSHRS cut-off values of TCGA and GSE44001 cohorts, patients were divided into high- and low-CSHRS groups. KM and log-rank analyses revealed that, in both cohorts, CESC patients had worse Overall survival (OS)in the high-CSHRS group (Figure 3E,F). The overall CSHRS (top panel), survival time (middle panel), and gene expression levels (bottom panel) for TCGA and GSE44001 datasets were shown in Figure 3G,H. Furthermore, CSHRS effectively predicted OS in the TCGA (AUC for 1-, 5-, and 7-year OSs: 0.847, 0.745, and 0.732, Figure 3I) and GSE44001 cohorts (AUC for 1-, 5-, and 7-year OSs: 0.696, 0.525, and 0.603, Figure 3J).

### 2.4. Patients with High-CSHRS Are Less Immune Infiltrated

**Figure 4:**
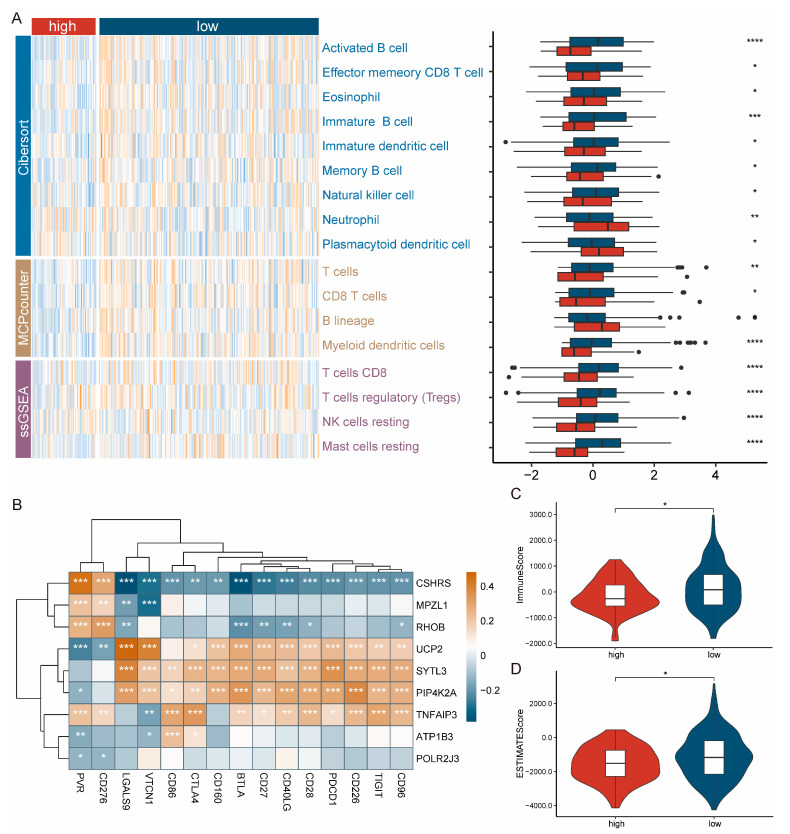
Patients with high-CSHRS are less immune-infiltrated. (**A**) Immune infiltrates analysis for CSHRS groups. (**B**) Correlation analysis between CSHRS and immune checkpoints. (**C**) Difference analysis for CSHRS groups in immune score. (**D**) Differences analysis for CSHRS groups in ESTIMATE score. *p* < 0.05 *, *p* < 0.01 **, *p* < 0.001 ***, *p* < 0.0001 ****.

Evidence shows that cellular and acellular components in the tumor microenvironment (TME) can reprogram tumor initiation, growth, invasion, and metastasis [32]. Considering the increasing significance of TME in cancer biology, we analyzed CSHRS grouping differences within the tumor microenvironment to clarify the biological mechanisms underlying the model. Firstly, the immune infiltration was estimated by ssGSEA, Cibersort, and MCPcounter, and the distribution differences between high- and low-CSHRS groups were investigated. As shown in a comprehensive heatmap (Figure 4A), the low-CSHRS group had a higher abundance of immune infiltrates, such as Activated B cells, T cells, and Mast cells resting. Subsequently, we conducted a correlation analysis between CSHRS and immune checkpoints [33]. The results show that CSHRS was negatively correlated with the expression of immune co-stimulatory signals, such as *CD27*, *CD28*, and *CD40LG*, as well as traditional immune checkpoints, including *CTLA4* and *PDCD*, and was positively correlated with *PVR* and *CD276* expression. These findings suggest that high-CSHRS patients may decrease anti-tumor immunity by inhibiting immune co-stimulatory signaling and promote tumor proliferation and metastasis by inhibiting T-cell activity through high expression of *PVR* and *CD276* genes (Figure 4B). Finally, ESTIMATE analysis demonstrated that the distributions of the immune score (Figure 4C) and ESTIMATE score (Figure 4D) were lower in high-CSHRS groups. In summary, high-CSHRS groups had less prominent immune infiltration and may exhibit higher immune response inhibition.

**Figure 5:**
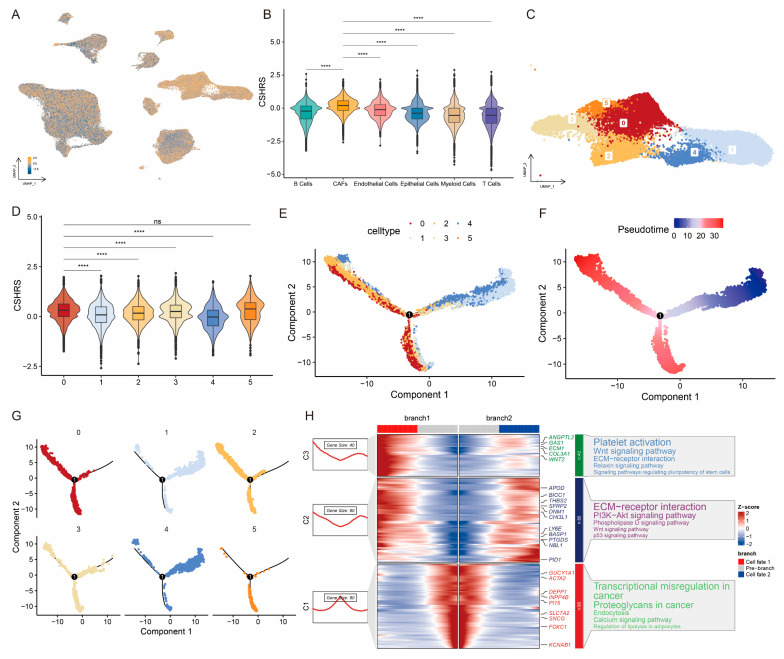
CSHRS reveals the differentiation process of CAFs cells. (**A**) Grouping cells by CSHRS. (**B**) Differences analysis of CSHRS in cell types. (**C**) UMAP plot showing the CAFs re-clustered. (**D**) Differences analysis of CSHRS in CAFs subtypes. (**E**) CAFs trajectory analysis colored by cluster. (**F**) CAFs trajectory analysis colored by Pseudotime. (**G**) CAFs trajectory analysis facet by cluster. (**H**) Heat map visualizing branching cell trajectories and gene dynamics in CAFs. *p* > 0.05 ns. *p* < 0.0001 ****.

To further investigate the role of the CSHRS in TME, we analyzed its impact from the scRNA-seq perspective. Firstly, we calculated the CSHRS for individual cells and observed that CSHRS were significantly higher in CAFs than in other cell types (Figure 5A,B). CAFs are a highly heterogeneous population of stromal cells, and hypoxia can stimulate them to secrete cytokines, chemokines, and growth factors that contribute to tumorigenesis, proliferation, and invasion [34,35,36,37,38]. Based on this finding, we focused on CAFs and re-clustered them into six subtypes using the FindClusters function with a resolution of 0.3 (Figure 5C). Then, we calculated the CSHRS of every subtype. Notably, CSHRS was significantly different among these subtypes, with clusters 0 and 5 showing the higher CSHRS and cluster 4 exhibiting the lower (Figure 5D). Using Monocle2, we inferred the potential developmental trajectory of CAFs (Figure 5E–G). Clusters 1 and 4 were identified at the starting point of differentiation, while clusters 0 and 5 were located at the terminal stage. Subsequently, we applied the branch expression analysis model (BEAM) to classify genes that exhibited significant changes at branch points (Figure 5H). Genes highly expressed at the differentiation starting point were primarily involved in transcriptional regulation, nutrient uptake, and energy metabolism processes, such as Transcriptional misregulation in cancer and Endocytosis [39,40]. Conversely, genes expressed at higher levels at the differentiation terminal were mainly associated with angiogenesis, DNA damage repair, proliferation, and metastasis in cancer, such as Wnt Signaling Pathway and p53 Signaling Pathway [41,42]. These findings suggest that CSHRS showed the differentiation trajectory of CAFs and was positively correlated with the degree of differentiation, thereby showing the adaptability of CAFs within the TME and their capacity to facilitate tumor progression.

### 2.5. Patients with High-CSHRS Have Increased Genomic Instability

**Figure 6:**
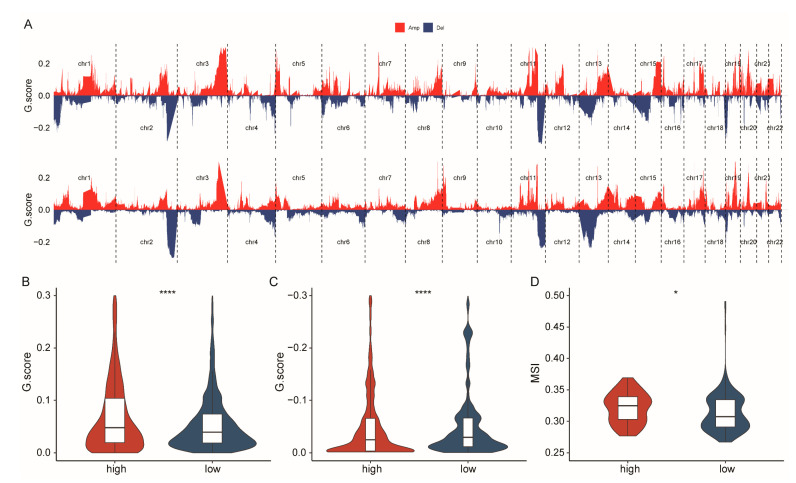
Patients with high-CSHRS have increased genomic instability. (**A**) CNV for the high-CSHRS group (top) and low-CSHRS group (bottom). (**B**) Difference analysis of copy number amplification in CSHRS groups. (**C**) Difference analysis of copy number deletion in CSHRS groups. (**D**) Difference analysis of MSI in CSHRS groups. *p* < 0.05 *. *p* < 0.0001 ****.

Genomic instability is a hallmark of cancer, driving tumorigenesis and evolution [43]. Therefore, we linked genomic instability with the CSHRS group to further clarify the biological mechanisms underlying the model. As shown in the Copy Number Variation (CNV) analysis (high-CSHRS: Figure 6A (top), low-CSHRS: Figure 6A (bottom), Amp: Figure 6B, Del: Figure 6C), a higher frequency of amplifications was observed in the high-risk group, while the low-risk group exhibited a higher frequency of deletions. As shown in the microsatellite instability (MSI) analysis (Figure 6D), the high-CSHRS group exhibited significantly higher MSI levels.

These findings reveal distinct mechanisms driving tumor progression in different CSHRS groups: in the high-CSHRS group, activation of oncogenes, combined with increased genomic instability, contributes to more aggressive tumor progression. In contrast, the low-CSHRS group maintains genomic stability by losing tumor suppressor genes or key regulatory genes, which fosters a more stable tumor environment and leads to a better prognosis.

### 2.6. Patients with High-CSHRS Benefited Less from Chemoradiotherapy

**Figure 7:**
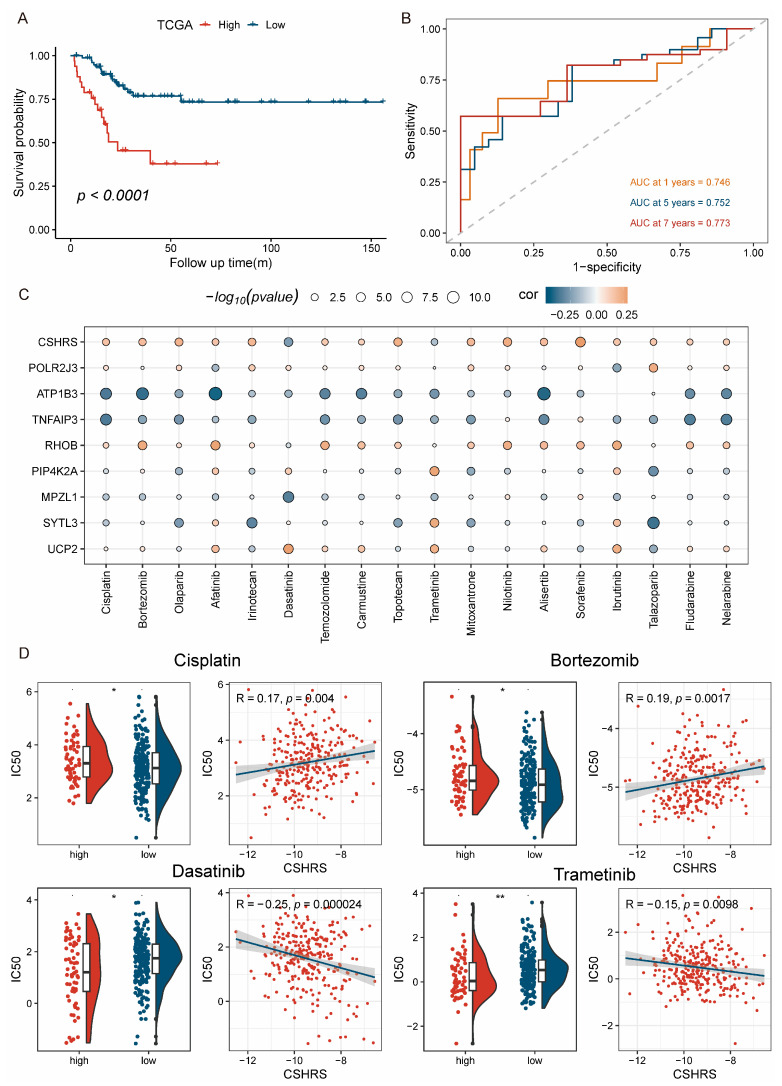
Patients with high-CSHRS benefited less from chemoradiotherapy. (**A**) Kaplan–Meier survival curves. (**B**) Time-dependent ROC curves analysis. (**C**) Correlation analysis between CSHRS, model genes, and drugs. (**D**) Correlation analysis and differences analysis of drug IC50 values in the low-CSHRS and high-CSHRS groups. *p* < 0.05 *. *p* < 0.01 **.

Currently, chemoradiotherapy remains the primary treatment method for advanced CESC patients, with cisplatin being one of the widely used drugs due to its proven efficacy in improving patient prognosis [44,45]. However, the heterogeneity among CESC leads to cisplatin resistance in some patients, negatively affecting prognosis. To analyze the predictive capability of CSHRS for chemoradiotherapy treatment outcomes, we first selected CESC patients from the TCGA who had exclusively received cisplatin-based therapy. A Kaplan–Meier survival curve analysis revealed that patients in the high-CSHRS group showed limited therapeutic benefit and relatively shorter progression-free survival (PFS) (Figure 7A). As shown in a time-dependent ROC curve analysis (Figure 7B), CSHRS functioned effectively in predicting 1-, 5-, and 7-year PFS (AUC for 1-, 5- and 7-year PFS: 0.746, 0.752, and 0.773, Figure 7B). These findings suggest that high-CSHRS patients have poor response to cisplatin treatment. To further explore therapeutic options, we first selected 18 commonly used chemotherapeutic agents and utilized the oncoPredict package to predict their efficacy in patients by estimating their respective IC50 values. The correlation and significance difference analysis between drug IC50 values and CSHRS are shown in Figure 7C,D. By analyzing the correlation between drug IC50 values and CSHRS, we evaluated drug sensitivity while also gaining insights into potential mechanisms of resistance through their association with the model genes. We observed that the IC50 values of Cisplatin and Bortezomib were higher, whereas those of Dasatinib and Trametinib were lower in the high-CSHRS group. These findings suggest that CESC patients with high-CSHRS were resistant to standard chemoradiotherapy regimens. However, they may be sensitive to two targeted therapies commonly used for chronic myeloid leukemia and melanoma. Therefore, Dasatinib and Trametinib have the potential as therapeutic options for CESC patients resistant to chemoradiotherapy.

### 2.7. Construction and Validation of the Nomogram Model

**Figure 8:**
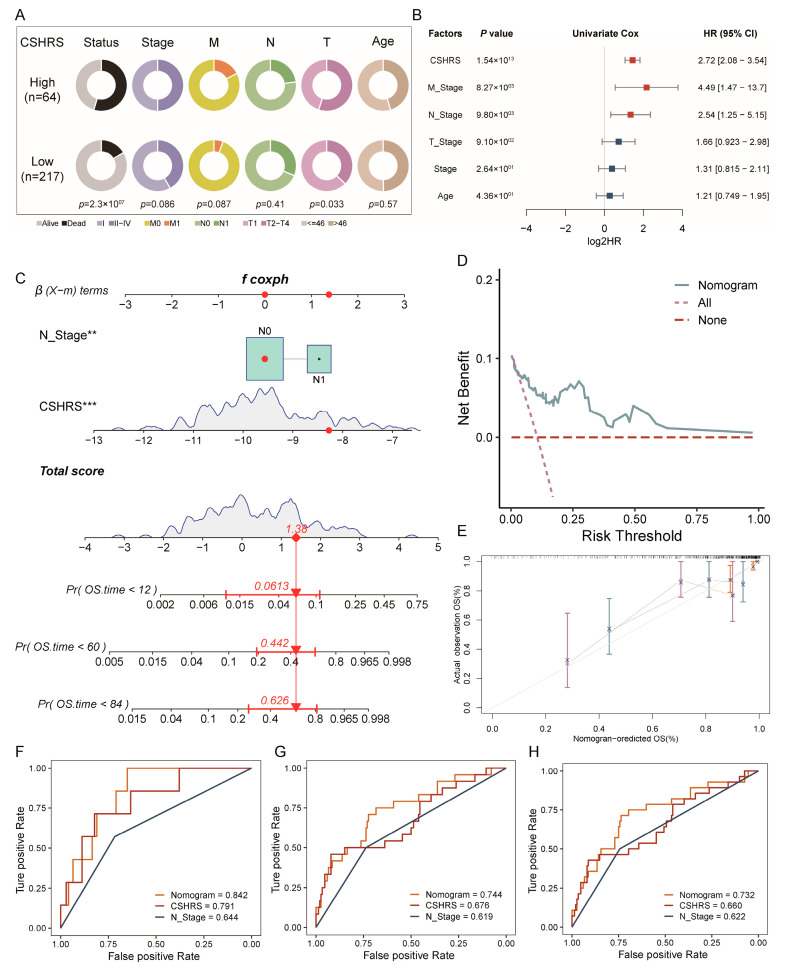
Construction and validation of the nomogram model. (**A**) Correlation analysis between CSHRS and clinicopathological features. (**B**) Univariate Cox regression analysis in clinicopathological features. (**C**) Nomogram for predicting the 1-, 5-, and 7-year OS. (**D**) DCA curve of the nomogram. (**E**) Calibration curves of the nomogram for predicting 1-, 5-, and 7-year OS. (**F**–**H**) ROC curves from 1 year (**F**), 5 years (**G**), and 7 years. (**H**) Nomogram diagram compared with individual factors accuracy.

Clinicopathological features reflect the physical conditions of patients and significantly impact prognosis. We constructed a nomogram to comprehensively evaluate the prognosis by combining CSHRS with clinicopathological features. Correlation analysis showed that CSHRS grouping was significantly correlated with the OS and T stage of patients (Figure 8A). In the low-CSHRS group, the proportion of early stages (T0, Stage I, M0) was higher, confirming the correlation between CSHRS and some clinical conditions (Figure 8B). Subsequently, we conducted univariate and multivariate Cox regression analyses to evaluate whether the CSHRS and various clinicopathological features are independent prognostic factors. The univariate Cox regression indicated significant associations with OS for the CSHRS, N stage, and M stage (Figure 8B). The multivariate Cox regression further identified the N stage and CSHRS as significant independent predictors of OS. Consequently, we integrated the CSHRS with the N stage to construct a predictive nomogram for assessing OS [41,42] (Figure 8C). The Decision Curve Analysis (DCA)curve illustrated that the nomogram’s curve deviated further from the two reference lines (Figure 8D). A calibration curve demonstrated the agreement between the nomogram-predicted OS estimates at 1, 5, and 7 years and the actual observed values (Figure 8E). The discriminative ability of the nomogram was compared to other individual factors using ROC curves for 1-, 5-, and 7-year survival. The nomogram had higher AUC values than any other factor (1 year: Figure 8F; 5 years: Figure 8G; 7 years: Figure 8H; AUC: 1 year: 0.842; 5 years: 0.744; 7 years: 0.732). These results confirm the accuracy of our proposed nomogram model.

## 3. Discussion

Hypoxia is a critical driver of accelerated cervical squamous cell carcinoma and endocervical adenocarcinoma (CESC)proliferation and has been strongly associated with poor prognosis and drug resistance [11,46]. Although some hypoxia-related gene signatures have been established for survival prediction, the single-cell RNA sequencing (scRNA-seq) mechanisms underlying hypoxia-associated prognosis in CESC remain undetermined and scRNA-seq is crucial for heterogeneity analysis [47,48]. Therefore, this study integrates scRNA-seq and bulk data for a comprehensive analysis of hypoxia signatures in CESC. We identified hypoxia-related gene signatures shared between scRNA-seq and bulk data and constructed a CESC-specific hypoxia-related score (CSHRS)risk model using machine learning algorithms. This model effectively stratified CESC patients into two distinct CSHRS groups, providing a novel reference for precision treatment strategies.

Comprehensive analysis at both scRNA-seq and bulk levels revealed that the prognostic differences between CSHRS groups are mainly attributed to differences in genomic instability and the immune microenvironment. Specifically, the high-CSHRS group showed higher genomic instability, characterized by increased microsatellite instability (MSI)scores and copy number amplification frequencies. These changes improve tumor adaptability and DNA damage repair capabilities, thereby promoting tumor proliferation [49,50]. Simultaneously, the high-CSHRS group showed a significant reduction in immune infiltration, with lower levels of T-cell and B-cell infiltration and increased cancer-associated fibroblasts (CAFs)infiltration. Further analysis showed that the CSHRS reveals CAFs’ differentiation trajectory, shifting from a normal metabolic state under low-hypoxia conditions to a tumor-associated malignant metabolic state in a high-hypoxia environment. Enrichment analysis showed that, as hypoxia in the tumor microenvironment aggravates, CAFs progressively adapt and promote tumor cell proliferation through mechanisms such as enhanced DNA repair capacity and angiogenesis induction [37,51]. Therefore, CSHRS can serve as an effective tool for identifying and monitoring the metabolic transitions of CAFs, providing novel strategies for the personalized treatment of patients.

Notably, CSHRS can also predict patients’ drug sensitivity, with its accuracy validated in Cisplatin sensitivity. Specifically, the high-CSHRS patients resisted DNA replication stress-inducing drugs, such as Cisplatin, Carmustine, Temozolomide, and Mitoxantrone. These drugs directly affect DNA integrity or target DNA polymerases and DNA damage-response proteins, leading to replication arrest [52,53]. The resistance may be attributed to the increased DNA damage-repair capacity in the high-CSHRS group, enabling the repair of chemotherapy-induced damage. In contrast, Dasatinib and Trametinib, as targeted therapies, inhibit tumor growth by blocking signal transduction and are less affected by DNA damage repair [54,55]. Thus, patients with high CSHRS may benefit from these treatments. Further research found opposite expression trends for the *PIP4K2A* and *RHOB* genes between drug-sensitive and drug-resistant groups. In drug-resistant groups, *PIP4K2A* expression negatively correlated with drug IC50 values, promoting cancer cell proliferation and survival through the activation of downstream pathways such as PI3K-AKT [56,57]. *RHOB* expression positively correlated with IC50 values, promoting the suppression of tumor growth by inducing G2/M cell cycle arrest and increasing apoptosis [58,59]. These findings highlight that high-CSHRS patients are resistant to DNA damage-inducing drugs but could benefit from therapies targeting alternative pathways.

There are several limitations in our study. The data utilized are retrospective, requiring validation with a prospective cohort. The efficacy of Dasatinib and Trametinib, which were identified as effective for high-CSHRS patients, requires experimental confirmation. Additionally, the resistance mechanisms of chemoradiotherapy drugs need further investigation through wet lab experiments.

In summary, this study integrates scRNA-seq and bulk data to construct a CSHRS risk model that serves as a valuable prognostic marker for CESC patients, allowing for more accurate clinical prognostic assessments and providing a new direction for personalized treatment.

## 4. Materials and Methods

### 4.1. Data Collection

We downloaded gene expression profiles and associated clinical data for cervical squamous cell carcinoma and endocervical adenocarcinoma (CESC) from multiple sources on 10 January 2024, including The Cancer Genome Atlas (TCGA) [60] (PanCancer Atlas) via cBioPortal (https://www.cbioportal.org/), Gene Expression Omnibus (GEO, https://www.ncbi.nlm.nih.gov/geo/) [61], and Genome Sequence Archive for Human (GSA-Human, https://ngdc.cncb.ac.cn/gsa-human/) [62]. Processed HRA004971 data are available at Science Data Bank (https://doi.org/10.57760/sciencedb.11624). TCGA included 281 patients with clinical follow-up information and 113 only cisplatin-treated patients, GSE44001 [63] included 300 patients, and HRA004971 included 14 patients and 130,316 cells. Hypoxia-related genes associated with the HALLMARK_HYPOXIA and the *HIF-1* signaling pathway were obtained on 15 January 2024 from MSigDB (https://www.gsea-msigdb.org/gsea/msigdb) [64] and Kyoto Encyclopedia of Genes and Genomes (KEGG, https://www.genome.jp/kegg/) [65], including 282 hypoxia-related genes.

The comprehensive mutation landscape of CESC patients was analyzed and visualized using the “maftools” package (v 2.18.0) [66]. The analysis included missense, silent, nonsense, frameshift/in-frame insertions and deletions, and interruptions, while synonymous mutations were excluded [67]. Copy Number Variation (CNV)analysis was conducted using GISTIC_2.0 on 16 March 2024 (https://www.genepattern.org/#).

### 4.2. scRNA-Seq Data Processing and Cell Annotation

The Seurat package (v 4.4.0) was used to analyze the single-cell RNA sequencing (scRNA-seq) dataset HRA004971. Firstly, quality control was applied to scRNA-seq data, retaining cells with more than 200 genes, unique molecular identifier counts between 1000 and 30,000, and a proportion of mitochondrial gene counts less than 10%. Secondly, the harmony function was used to eliminate the batch effects. Dimensionality reduction and unsupervised clustering were applied based on a resolution of 0.1 and were annotated cells using marker genes [68].

### 4.3. Calculation of the Hallmarks Score

The levels of cancer-related hallmarks proposed by Hanahan [20], such as “Epithelial–mesenchymal transition (EMT)”, “Fatty acid metabolism (FAM)”, and “Hypoxia”, were quantified using a single-sample gene set enrichment analysis (ssGSEA, v 1.50.5) [69] algorithm, based on the corresponding gene sets obtained on 17 January 2024 from the MSigDB (https://www.gsea-msigdb.org/gsea/msigdb).

### 4.4. Detection and Selection of CESC-Specific Hypoxia-Responsive Genes

Hypoxia gene sets from databases and the literature for prognostic analysis may lack tumor specificity. Therefore, we integrated bulk and scRNA-seq data to identify CESC-specific hypoxia-related genes based on current knowledge and CESC expression profiles. Firstly, a Weighted Gene Correlation Network Analysis (WGCNA, v 1.72-1) [70] was performed to identify genes associated with the hypoxia phenotype in bulk data of CESC patients. The soft thresholding parameter (β) was determined to be 0.8. Pearson correlation analysis was applied to evaluate the association between each gene module and identify the most common correlation module with hypoxia. Subsequently, the hypoxia score was calculated for each cell using the AUCell package (v 1.22.0) in scRNA-seq data. AUCell assesses the enrichment of a gene set within individual cells by analyzing single-cell expression data. During computation, it first ranks all genes within each cell based on their expression levels. It then identifies the positions of genes from the hypoxia gene set within this ranking and calculates the AUC to quantify gene set activity. A higher AUC value, referred to as the hypoxia score, indicates greater gene set activity, reflecting stronger expression within the cell. The high- and low-hypoxia cells were grouped by the median of the hypoxia score and using the FindMarkers function (v 4.4.0) to identify differentially expressed genes (*p* < 0.05 & logFC > 0.25). The intersection between the model genes and differentially expressed genes was deemed as the CSHRGs.

### 4.5. Construction of the CSHRS Risk Model

Firstly, the association between CSHRGs expression and overall survival (OS) was assessed using a univariate Cox regression analysis (*p* < 0.05). Subsequently, the least absolute shrinkage and selection operator (LASSO) regression was applied to evaluate the combined effects of genes and identify significant factors from the univariate analysis. Finally, to construct the model for independent prognostic markers, a stepwise multivariate Cox regression analysis was conducted, resulting in the development of the CSHRS risk model.

$$CSGRS=\sum_{i}^{n}{coef}_{i}\times {Exp}_{i}$$

The optimal cut-off values for the CSHRS were determined using the surv_cutpoint function. Patients from the TCGA training set, along with the GSE44001 validation sets, were subsequently classified into high and low CSHRS groups. Survival analyses and log-rank tests were performed using the survival package to evaluate OS. Risk scatter plots were generated to visualize the distribution, survival time, and survival status of patients in CSHRS groups, using the TimeROC function (v 0.4) to evaluate the AUC values of patient 1-, 5-, and 7-year dependencies.

### 4.6. Immune Infiltration Analysis

Immune-related genes were obtained from the publication PMID:28052254 [71]. Immune cell infiltration levels were assessed using the ssGSEA (v 1.50.5), Cibersort (v 1.03) [72], and MCPcounter (v 1.2.0) [73] algorithms. Immune scores for each patient were calculated using the ESTIMATE package (v 1.0.13) [73].

### 4.7. Construction of the Nomogram Model

The RMS package (v 6.8-1) was used to construct a clinical prognostic nomogram incorporating CSHRS and clinicopathological features significantly associated with OS (*p* < 0.05). Decision Curve Analysis (DCA) and calibration curves were generated, comparing the predicted OS with the observed OS to evaluate the nomogram’s predictive performance.

### 4.8. Statistical Analysis

All data processing and analyses in this study were conducted using R software (v 4.4.1). The statistical significance of normally distributed continuous variables between two groups was assessed using an independent Student’s t-test. For non-normally distributed variables, differences were analyzed using a Wilcoxon rank-sum test. Survival analyses were conducted using the survival package (v 3.6.4) in R, employing Kaplan–Meier survival curves to illustrate survival differences. A log-rank test was employed to assess the significance of differences in survival times between the two patient groups. Univariate and multivariate Cox analyses were also performed using the survival package (v 3.6.4). All statistical *p*-values were two-sided, with *p* < 0.05 considered to be statistically significant.

## Data Availability

The data that supports the findings of this study are available from public databases.

## Supplementary Materials

The following supporting information can be downloaded at: https://www.mdpi.com/article/10.3390/ijms26031362/s1.
