# Supplementary material for: Integrating Hypoxia Signatures from scRNA-seq and Bulk Transcriptomes for Prognosis Prediction and Precision Therapy in Cervical Squamous Cell Carcinoma and Endocervical Adenocarcinoma
Source: Int J Mol Sci. 2025 Feb 6;26(3):1362. doi: 10.3390/ijms26031362 (PMC11818358; doi:10.3390/ijms26031362)
Supplement: Supplementary file 1 [file ijms-26-01362-s001.zip › Supplementary Files/Supplementary.pdf]

Supplementary Figure S1 Research Process Flowchart

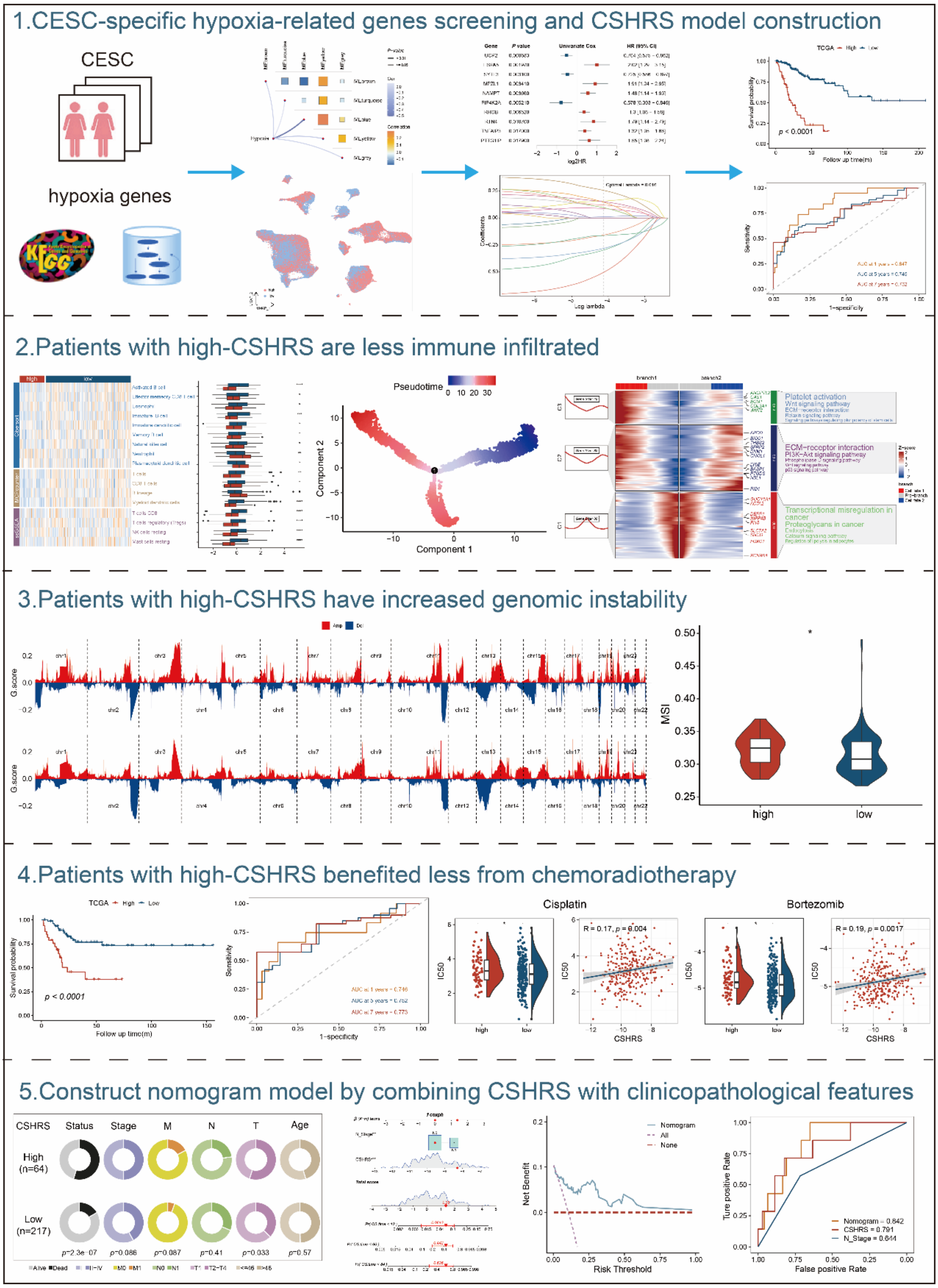

Supplementary Figure S1 Research Process Flowchart. Firstly, the CESC-specific hypoxia gene sets shared between scRNA-seq, and bulk data were identified using WGCNA and FindMarkers. A CSHRS risk model was constructed using LASSO and Cox regression analyses based on these genes. The prognostic differences were explored from immune infiltration, mutations, and drug

resistance perspectives. Finally, a nomogram model was constructed by integrating clinicopathological features to facilitate precision treatment for CESC.

Supplementary Table: Supplementary table on tumor marker levels. Table S1: Table of tumor marker genes. Table S2: Table of tumor marker levels.
